# Determination of water in organic solvents by ligand-exchange reaction of Tris(2-methyl-8-quinolinolato)indium(III) complex

**DOI:** 10.1007/s44211-026-00911-3

**Published:** 2026-04-20

**Authors:** Nanami Watanabe, Nobuo Uehara, Arinori Inagawa

**Affiliations:** School of Engineering, Utsunomiya University, 7-1-2 ,Yoto, Utsunomiya, Tochigi, 321- 8585 Japan

**Keywords:** Water determination, Tris(2-methyl-8-quinolinolato)indium(III), Fluorescence sensor, Organic Solvent, Ligand-exchange reaction

## Abstract

The water content of organic solvents was determined by the ligand dissociation of a tris(2-methyl-8-quinolinolato)indium(III) complex. Water in the organic solvent reacts with the indium complex to induce a ligand-exchange reaction. The fluorescence of deprotonated 2-methyl-8-quinolinol disappears when the ligand-exchange reaction forms a neutral molecule; thus, the complex can serve as a turn-off water-detection sensor. We measured the water content of various organic solvents, including ethyl acetate, 1-octanol, and ethanol. The indium complex was dissolved in a water-containing organic solvent, the fluorescence of the 8-quinolonolate ion was measured, and determination curves were constructed based on the decrease in fluorescence intensity. To demonstrate the versatility of the present method, we measured the water content of alcoholic drinks. These results are consistent with the data obtained by Karl Fischer (KF) titration. Although the limits of detection and quantification were not as low as those of the KF titration, the simple experimental setup and procedure of this method are favorable for its application.

**Graphical abstract:**

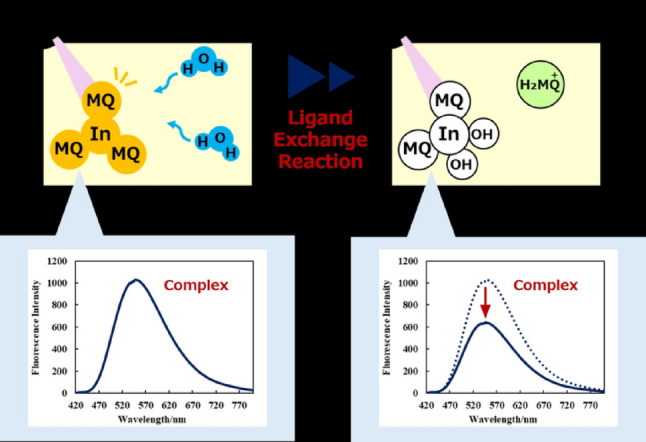

**Supplementary Information:**

The online version contains supplementary material available at 10.1007/s44211-026-00911-3.

## Introduction

Trace water in organic solvents can critically affect chemical reactions, leading to catalyst deactivation, undesired side reactions, corrosion, and the deterioration of product quality [1]. Therefore, a reliable and simple method for the determination of water content at the ppm or sub-ppm level is essential, particularly in the chemical industry for handling moisture-sensitive materials, in petroleum and oil refining processes and fuel production, and for the quality control of ethanol in the solvent and alcoholic beverage industries [2]. 

The Karl Fischer (KF) titration method, originally developed by Karl Fischer in 1935, is one of the most widely used techniques for water determination. Both volumetric and coulometric KF titrations enable highly accurate quantification of water [3, 4]. However, this method involves multiple competing equilibrium and side reactions depending on the measurement conditions, which complicates the analytical system and may cause interference from coexisting substances [5]. For example, aldehydes and ketones can react with methanol to form acetals, reducing agents may consume iodine, and acidic or basic impurities may disturb the reaction equilibrium. Additionally, KF titration requires dedicated instrumentation, careful handling of the reagents and waste solutions, and strict control to avoid moisture contamination, which can lead to operational complexities and errors [6]. Therefore, the development of simpler and reagent-free alternative methods is desirable for routine or on-site analysis.

In recent years, alternative approaches for water determination based on infrared spectroscopy, electrochemical sensing, and optical methods have been explored [7, 8]. Among these, fluorescence-based methods have attracted considerable attention owing to their high sensitivity, rapid response, low sample consumption, and nondestructive nature [9]. However, many previously reported fluorescence-based water sensors suffer from limitations such as complicated probe synthesis, restricted applicability to specific solvents, insufficient stability, and poor reproducibility, hindering their widespread practical use [10]. 

In this note, a novel method for the determination of trace amounts of water in organic solvents using an indium(III)–2-methyl-8-quinolinol complex (In(MQ)₃) as a turn-off-type fluorescent probe is described. In this study, we focused on the ligand-exchange reaction between In(MQ)₃ and water and developed a method based on fluorescence quenching for the determination of trace water in organic solvents. The coordination of water molecules to the indium center induces a decrease in the concentration of the fluorescent In(MQ)₃ complex, leading to reduced fluorescence intensity. The quantitative determination of trace water is enabled by correlating this decrease with the water concentration. Calibration curves were constructed for several organic solvent systems, and the applicability of the proposed method to real samples was evaluated. Furthermore, the analytical performance of the method was assessed by comparison with conventional techniques. The developed method, which can be implemented using a conventional fluorescence spectrophotometer, offers a simple and practical alternative for the analysis of trace amounts of water in organic solvents.

## Experimental section

### Chemicals

Indium(III) nitrate and 2-methyl-8-quinolinol were purchased from Tokyo Chemical Industry (Tokyo, Japan). Ammonium acetate, hydrochloric acid, calcium carbonate, sodium hydroxide, 1-octanol, dichloromethane, hexane, and anisole were purchased from Kanto Chemical Co. (Tokyo, Japan). All aqueous solutions were prepared using ultrapure water obtained from a Milli-Q system (Merck, USA).

## Synthesis of in(MQ)_3_

Based on a previously reported method for the synthesis of typical 8-quinolinol complexes [11], In(MQ)_3_ was synthesized as follows: indium(III) nitrate (3.5450 g) and 2-methyl-8-quinolinol (1.9890 g) were individually dissolved in 1.0 M hydrochloric acid. The two solutions were then mixed, and the pH was set to 6–8 by adding ammonium acetate solutions. The complex was then precipitated, and the precipitated complexes were filtered and washed with hexane. The pure complex was recrystallized from hexane and characterized using absorption spectroscopy, elemental analysis, and electrospray ionization mass spectrometry (ESI-MS).

## KF titration for determination of water content of organic solvents

A KF titration was performed to determine the amount of free water present in the organic solvent. An AQV-200 automated titrator (Hiranuma, Japan) with Hydronal Composite 5 titrant (Honeywell, Fluka, Germany) was used. An aliquot of the organic solvent-containing water was dissolved in dry methanol and titrated.

## Spectrometric measurement

The prepared samples were poured into quartz cuvettes (optical path length: 1.0 cm). Fluorescence spectra were measured using an FP-8300 fluorometer (JASCO, Japan), and absorption spectra were measured using a U-650 spectrometer (JASCO, Japan).

## Preparation of sample and determination curve

Ethanol was mixed with a certain amount of water to prepare a water-containing sample. For the ethyl acetate and 1-octanol samples, a given amount of the water-saturated organic solvent was mixed with the dried solvent. Dried organic solvents were prepared by mixing with calcium carbonate and stirring overnight. The water content of the organic solvents was determined by KF titration.

For the spectrophotometric detection of the water content, 1 mL of In(MQ)_3_ dissolved in ethanol or dichloromethane was mixed with 4 mL of the organic solvent sample, and the prepared samples were analyzed spectroscopically. The fluorescence intensity of organic solvents with known water content was then plotted against the water content determined by KF titration to obtain the determination curve.

### Determination of water content of alcoholic sample

For real sample detection, we employed a commercially available *shochu* liquor. A *shochu* sample (0.5 mL) was mixed with dried ethanol (3.5 mL) and In(MQ)_3_ solution (1.0 mL), and the samples were analyzed using a fluorometer. The water content of *shochu* was determined from the fluorescence intensity and determination curve. The water content of the organic solvents was determined by KF titration.

## Results and discussion

### Ligand-exchange reaction of In(MQ)_3_ with water in organic solvents

The ligand-exchange reaction of In(MQ)_3_ with water molecules was examined in an organic solvent. Previous studies have revealed that (i) indium can form complexes with hydroxide ions [12, 13] and (ii) In(MQ)_3_ undergoes a ligand-exchange reaction with silanol groups [14–16]. Rane et al. studied the ligand-exchange reaction of In(MQ)_3_ with hydroxide ions in aqueous solution and determined the stepwise equilibrium constants. However, the present study aimed to determine the water content of organic solvents; therefore, we examined the ligand-exchange reactions in various organic solvents.

**Fig. 1:**
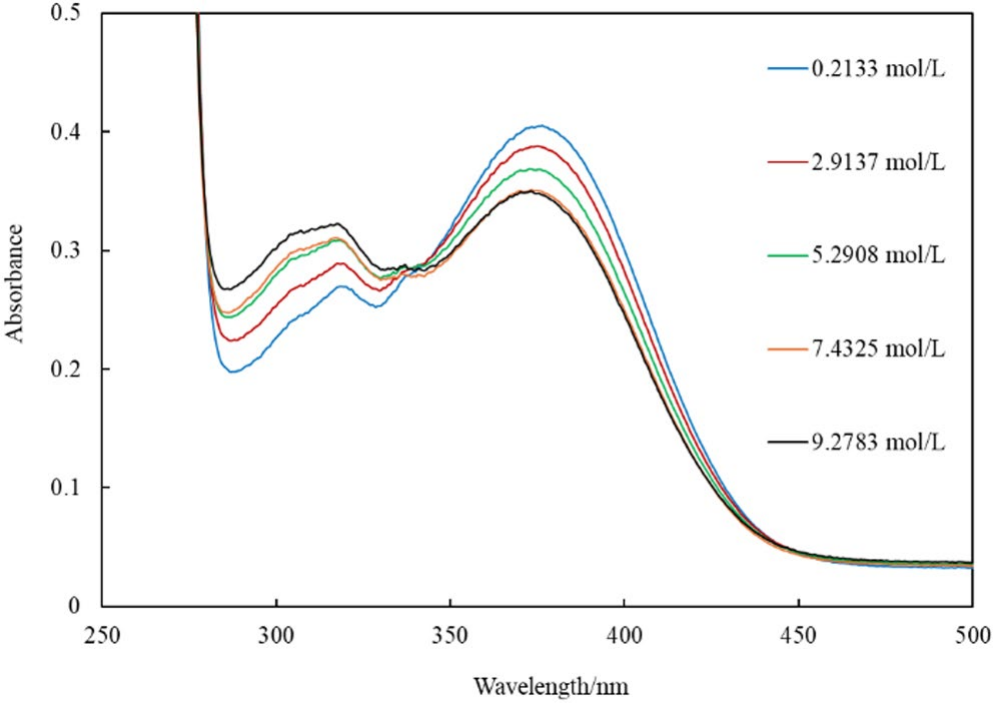
Absorption spectra of In(MQ)_3_ in water-containing ethanol with different concentrations of water

Figure 1 shows the absorption spectra of In(MQ)_3_ in water-containing ethanol. The water content of the solvent was measured by KF titration, and the results are summarized in Table S1. Upon increasing the water concentration, the absorption in the 300–330 nm region increased owing to the liberated ligand, whereas the absorption band at 350–400 nm decreased, which was attributed to the coordination of the quinolinolate ion to indium (III). This behavior suggests that the ligand was liberated into the organic solvent via a ligand-exchange reaction with water molecules. As previously reported, quinolinol exists in a protonated state [17]. For quantitative evaluation, the changes in the concentrations of In(MQ)_3_ and H_2_MQ^+^ with increasing water concentration were examined. Figure S1 shows the determination curves of In(MQ)_3_ and H_2_MQ^+^ in ethanol. The non-zero intercept observed in the determination curves is attributed to the overlap of absorbance from coexisting species because peak deconvolution was not applied, and the total absorbance change was directly correlated with concentration. Using this curve, the concentration of each solution was calculated, and the equilibrium constant was determined. Based on the changes in the concentration of each species in the solution system, the overall reaction can be described as follows:

In(MQ)_3_ + 4H_2_O ⇄ [In(MQ)(OH)_4_]^2−^ + 2H_2_MQ^+^ (1).

Although the stepwise formation of intermediates or bis complexes cannot be completely excluded, the spectroscopic behavior observed within the investigated concentration range was satisfactorily described by a single-equilibrium approximation. Therefore, for simplicity, the present analysis considers a system using an apparent overall equilibrium constant. The concentration equilibrium constant for this reaction is expressed as:

2 $$\:K=\frac{\left[\mathrm{I}\mathrm{n}\left(\mathrm{M}\mathrm{Q}\right){\left(\mathrm{O}\mathrm{H}\right)}_{4}\right]{\left[{\mathrm{H}}_{2}\mathrm{M}\mathrm{Q}\right]}^{2}}{\left[\mathrm{I}\mathrm{n}{\left(\mathrm{M}\mathrm{Q}\right)}_{3}\right]{\left[{\mathrm{H}}_{2}\mathrm{O}\right]}^{4}}$$

To determine *K*, this equation can be rearranged as follows:

$$\:\mathrm{l}\mathrm{o}\mathrm{g}\frac{\left[\mathrm{I}\mathrm{n}\left(\mathrm{M}\mathrm{Q}\right){\left(\mathrm{O}\mathrm{H}\right)}_{4}\right]}{{\left[{\mathrm{H}}_{2}\mathrm{O}\right]}^{4}}=\mathrm{l}\mathrm{o}\mathrm{g}K-\mathrm{l}\mathrm{o}\mathrm{g}\frac{{\left[{\mathrm{H}}_{2}\mathrm{M}\mathrm{Q}\right]}^{2}}{\left[\mathrm{I}\mathrm{n}{\left(\mathrm{M}\mathrm{Q}\right)}_{3}\right]}$$

**Fig. 2:**
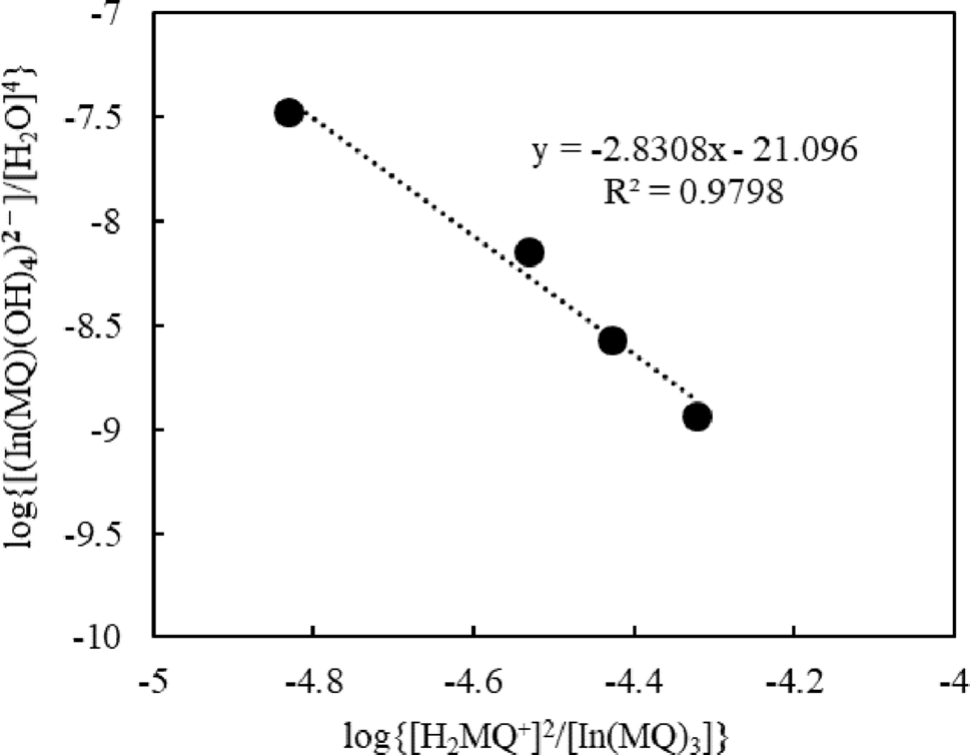
Plot of log{[In(MQ)(OH)_4_^2−^]/[H_2_O]^4^}versus log{[H_2_MQ⁺]^2^/[In(MQ)_3_]} for the ethanol samples

The equilibrium concentration of In(MQ)_3_ and [In(MQ)(OH)_4_]^2–^ can be estimated spectroscopically, whereas that of water can be determined by KF titration. The concentrations of In(MQ)₃ and H₂MQ⁺ were determined from their respective calibration curves shown in Fig. S1. The concentration of [In(MQ)(OH)₄]²⁻ was estimated by mass balance from the initial concentration of In(MQ)₃ and the amount of dissociated ligand. Figure 2 shows the plot used to determine *K* according to Eq. (3). Based on the y-axis intercept, log*K* is estimated as − 21.1. The obtained log*K* values should be regarded as semi-quantitative parameters rather than rigorous thermodynamic constants because they were estimated from limited concentration ranges. Nevertheless, these values are useful for comparing the solvent-dependent tendencies of the ligand-exchange reactions. These results indicate that the equilibrium is strongly influenced by solvent polarity and proticity.

**Fig. 3:**
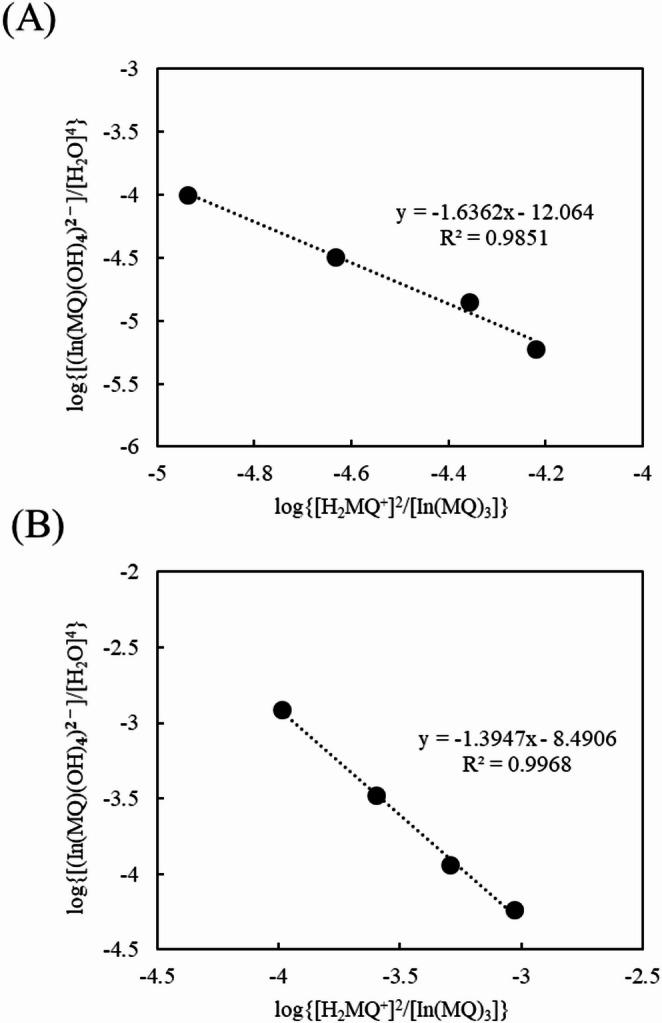
Plots of log{[(In(MQ)(OH)_4_)_2_-]/[H_2_O]_4_} versus log{[H_2_MQ⁺]_2_/[In(MQ)_3_]} in **A** 1-octanol and **B** mixture of ethyl acetate and dichloromethane with different water concentrations

While ethanol is a relatively hydrophilic organic medium, trace water analysis is important for less hydrophilic and hydrophobic solvents. In this study, we examined ligand-exchange reactions of In(MQ)_3_ in various solvents. Since only a limited number of solvents can dissolve In(MQ)_3_, we used 1-octanol and a mixture of ethyl acetate and dichloromethane (EA/DCM). The equilibrium analysis is similar to that described above for ethanol. Figure S2 shows a series of absorption spectra of In(MQ)_3_ and H_2_MQ^+^ in 1-octanol and EA/DCM with increasing water concentration. The water concentration was determined by KF titration and the results are summarized in Table S1. The change in absorbance indicates that the ligand-exchange reaction of In(MQ)_3_ was similar to that of ethanol. A determination curve was prepared for each solvent, as shown in Fig. S3, and the concentration changes of In(MQ)_3_ and H_2_MQ^+^ with varying initial water concentrations were determined. As described above, the concentration equilibrium constant, *K*, was estimated by plotting log{[In(MQ)(OH)_4_^2−^]/[H_2_O]^4^}versus log{[H_2_MQ⁺]^2^/[In(MQ)_3_]}, as shown in Fig. 3, with the y-axis intercepts indicating the log*K* values. Table 1 summarizes the log*K* values calculated for each organic solvent. It is observed that the log*K* values are affected by the solvent and the differences between the equilibrium constants are attributed to the differences between the dielectric constants and functional groups of the solvents [18, 19]. In ethyl acetate, which is an aprotic solvent, water molecules and dissociated OH⁻ ions are not effectively stabilized by the solvent, facilitating the coordination of OH⁻ to the central indium(III) metal ion of the complex. As a result, the ligand-exchange reaction is promoted. By contrast, in highly polar and protic solvents such as ethanol, water molecules and dissociated OH⁻ ions are stabilized by the solvent, thereby suppressing the coordination of OH⁻ to the indium(III) ion. Consequently, the ligand-exchange reaction is hindered. Busch et al. reported that for neutral acids such as alcohols and carboxylic acids, the p*K*_a_ values are lower in solvents with high ability to stabilize the conjugate base through dipole–dipole interactions and hydrogen bonding than in organic solvents with low polarity and aprotic character and limited ability to stabilize the conjugate base [20]. These results consistent with the common concept that solvent-induced stabilization of chemical species affects equilibrium reactions which thus supports the solvent effects on the equilibrium constants observed in the present study. Furthermore, the water concentration at equilibrium in water-saturated 1-octanol is 2.21 mol L⁻¹, whereas that in water-saturated ethyl acetate is 1.68 mol L⁻¹, while ethanol is completely miscible with water. These results reflect the differences in the stability of water molecules and OH⁻ ions in each organic solvent and provide further evidence for the solvent effects observed from the determined equilibrium constant values.

**Table 1:** Determined equilibrium constants of the ligand-exchange reaction in various organic solvents

log K		
Ethanol	Ethyl acetate	1-Octanol
–21.10	–8.491	–12.06

### Fluorescence properties of In(MQ)_3_ coexisting with water in organic solvent

To ensure quantitative fluorescence measurements, the absorbance of all samples at the excitation wavelength was kept below 0.05 by adjusting the concentration, thereby minimizing inner-filter effects and self-absorption. Fluorescence spectra were recorded at an excitation wavelength of 380 nm.

As described in the previous section, the water concentration of each sample used for calibration is summarized in Table S1. Ethanol was used as the solvent for the stock complex solution and dichloromethane was used for the ethyl acetate and 1-octanol systems. Because the complex is not soluble in 1-octanol alone, dichloromethane (1 mL), which can dissolve the complex, was added to both the stock solution and samples.

**Fig. 4:**
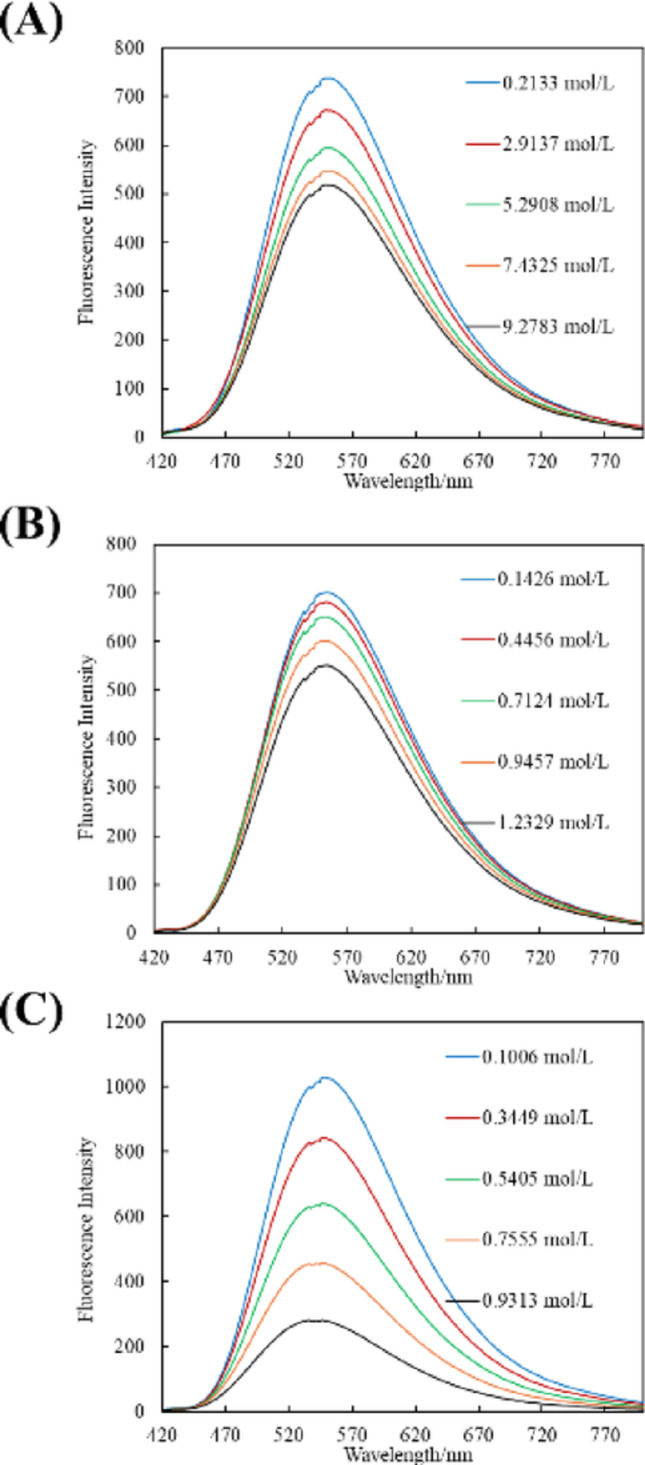
Fluorescence intensity of In(MQ)_3_ coexisting with water in **A** ethanol, **B** a mixture of 1-octanol and dichloromethane, and **C** a mixture of ethyl acetate and dichloromethane. Wavelength of the excitation light: 380 nm

**Fig. 5:**
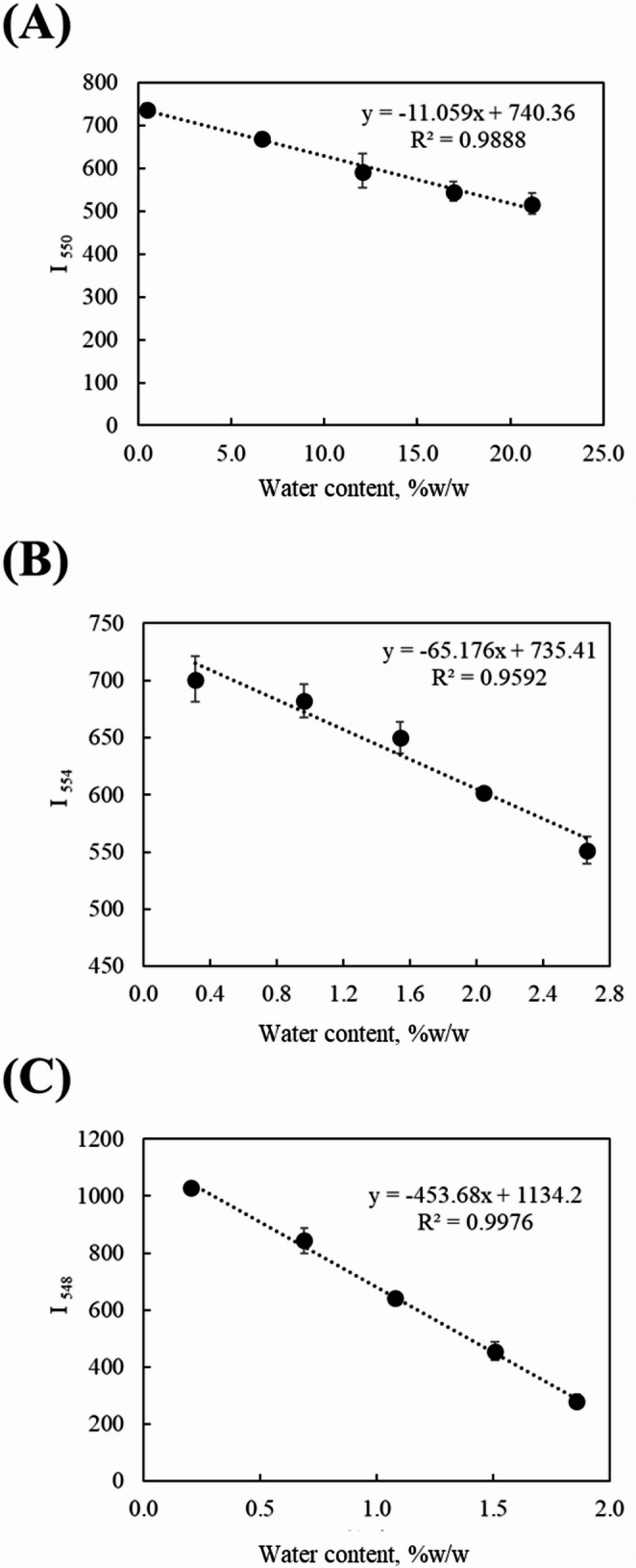
Calibration curve for water content determination using fluorescence intensity of In(MQ)_3_ in **A** ethanol, **B** mixture of 1-octanol and dichloromethane and (C) mixture of ethyl acetate and dichloromethane

The fluorescence spectra of the solutions after the reaction are shown in Fig. 4. A solution shaken for 40 min in a dry organic solvent was used as a blank for comparison. As shown in Fig. 4, the fluorescence intensity of the complex decreases with increasing water concentration in the solution. Based on the obtained fluorescence intensities, calibration curves of fluorescence intensity versus water concentration were constructed for each organic solvent sample. The fluorescence intensities at 550, 548, and 554 nm, corresponding to the main emission peaks of the complex in ethanol, ethyl acetate, and 1-octanol, respectively, were used to construct calibration curves by plotting them against the water content; the results are presented in Fig. 5. High R² values were obtained for all organic solvent samples, indicating good linearity and demonstrating that the calibration curves were suitable for quantitative analysis.

The limits of detection (LOD) and limits of quantification (LOQ) determined from the calibration curves are summarized in Table 2. The detection sensitivities of the ethanol and 1-octanol systems were slightly lower than that of the KF titration method employed in this study. In contrast, an LOD comparable to that of the KF titration method was obtained for the ethyl acetate system. Furthermore, the detection sensitivity of the present method was lower than that of the method reported by Yu et al. using lanthanide-doped upconversion nanoparticles for water determination in organic solvents [21]. However, the LOD achieved for the ethyl acetate system was comparable to that of the method using 4-(pyridine-2-yl)-3 H-pyrrolo[2,3-*c*]quinoline for water determination in organic solvents reported by Pawer et al. [22]

**Table 2:** LOD and LOQ of the fluorescence-based water determination and Karl-Fisher (KF) titration

Ethanol				1-octanol				Ethyl acetate			
This method		KF titration		This method		KF titration		This method		KF titration	
LOD, %w/w	LOQ, %w/w	LOD, %w/w	LOQ, %w/w	LOD, %w/w	LOQ, %w/w	LOD, %w/w	LOQ, %w/w	LOD, %w/w	LOQ, %w/w	LOD, %w/w	LOQ, %w/w
3.1	10.3	0.316	1.05	0.922	3.07	0.0074	0.0247	0.0793	0.264	0.0153	0.0511

### Real sample experiment

The water content of the real samples was determined using the calibration curves for water determination established as described above. Ethyl acetate with unknown water content and a commercially available barley *shochu* alcoholic beverage were examined as real samples.

**Fig. 6:**
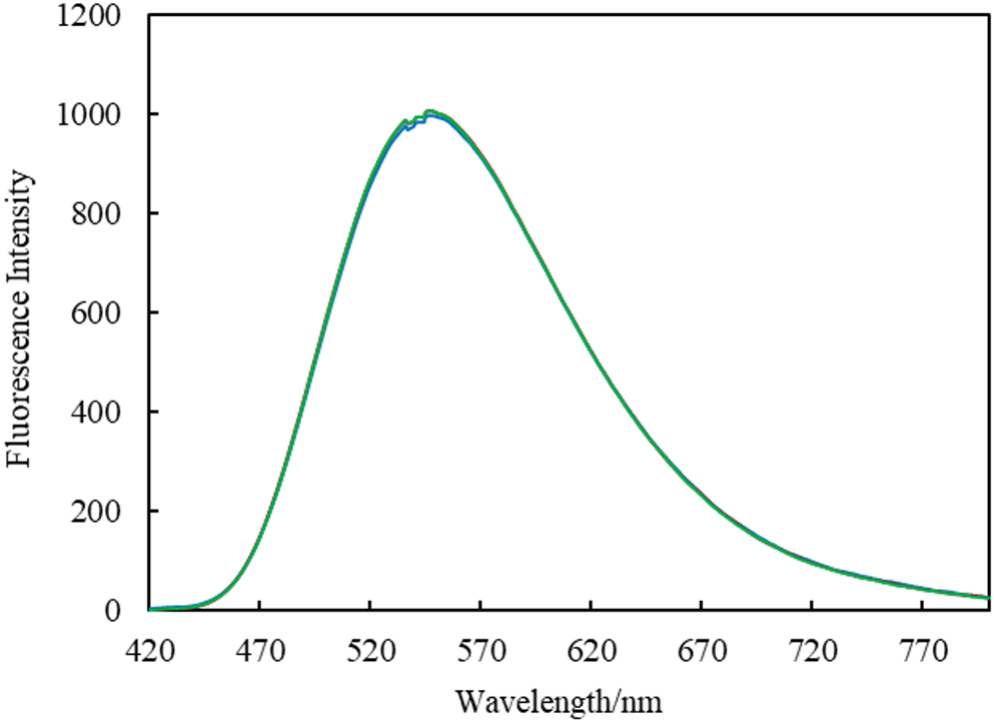
In(MQ)_3_ fluorescence spectra of ethyl acetate with unknown water concentration prepared by exposure to ambient environment. Wavelength of the excitation light: 380 nm

The fluorescence spectrum of ethyl acetate with unknown water content is shown in Fig. 6. In this spectrum, the fluorescence intensity at 548 nm, which corresponds to the main emission peak of the complex in ethyl acetate, was used to calculate the water concentration based on the calibration curve constructed in the previous section, with the results summarized in Table 3. For comparison, water concentration determined using the KF titration method, which is widely used for water determination, are also presented. A *t*-test was performed for the water concentrations obtained using the two methods, and no significant difference was observed between the present method and the KF titration method (*t*(2) = 1.51, 95% CI). These results demonstrate that the proposed method can accurately quantify the water content of ethyl acetate.

**Fig. 7:**
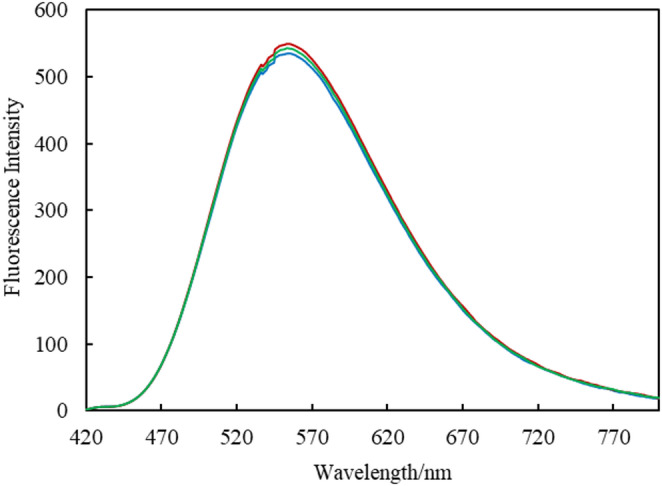
In(MQ)_3_ fluorescence spectra of the Japanese liquor sample used to determine its water content. Wavelength of the excitation light: 380 nm

The fluorescence spectrum of the barley shochu “Hon-kaku Shochu Hakata no Hana” diluted with ethanol, is shown in Fig. 7. In this spectrum, the fluorescence intensity at 550 nm, which corresponds to the main emission peak of the complex in ethanol, was used to calculate the water concentration based on the calibration curve constructed, as described in the previous section. The results are summarized in Table 4, along with the water concentrations determined using the KF titration method. A *t*-test comparing the results obtained using the two methods revealed no significant differences between the proposed method and the KF titration method (*t*(3) = 0.00417, 95% CI). These results indicate that the present method is also applicable for accurately determining the water content of commercially available alcoholic beverages. Furthermore, by employing a calibration curve covering a higher concentration range than that constructed in this study, it was confirmed that alcoholic beverages with higher water content than previously determined could also be analyzed, obtaining water concentrations that were not significantly different from those obtained by the KF titration method.

From these results, it is concluded that the present method is applicable to the determination of water content of organic solvents in which In(MQ)₃ is soluble. In addition, even for organic solvents in which In(MQ)₃ is insoluble, the present method can be applied by preparing calibration curves through the addition of a miscible organic solvent in which In(MQ)₃ is soluble. Although water content determination can also be achieved by absorption spectroscopy, the present fluorescence-based method offers advantages, such as higher sensitivity, rapid response, simple operation, and minimal reagent consumption, making it suitable for routine and portable analysis.

**Table 3:** Comparison of the results of the real sample experiment obtained by the present method and KF titration for the ethyl acetate sample

Water concentration, %w/w	
Present method	KF
0.2869 ± 0.0051	0.2751 ± 0.0047

**Table 4:** Comparison of the results of real sample experiment obtained by the present method and KF titration for the Japanese liquor sample

Water concentration, %w/w	
Present method	KF
11.4035 ± 0.0870	11.4041 ± 0.1124

## Conclusion

A fluorescence-based method for the determination of water content of organic solvents was developed by utilizing the ligand-exchange reaction between In(MQ)₃ and water. The coordination of hydroxide ions to the indium(III) center induces fluorescence quenching of the complex, enabling the quantitative evaluation of water concentration. Good linearity was obtained for the calibration curves constructed using ethanol, ethyl acetate, and 1-octanol, and the solvent-dependent behavior was attributed to the differences in solvent polarity and proticity. The analytical performance of the proposed method was comparable to that of the KF titration method, particularly for ethyl acetate. The applicability of the method was confirmed through analysis of a real ethyl acetate sample and a commercially available alcoholic beverage, which showed no significant differences between the water concentration values obtained by the proposed method and those obtained by KF titration. Furthermore, the method can be extended to solvents in which In(MQ)₃ is insoluble by adding a suitable miscible solvent. These results demonstrate that the proposed fluorescence quenching approach is a simple and versatile alternative for determining the water content in organic solvent systems. Possible interference from other coordinating impurities or pH-dependent species should also be considered because such components may compete with water for coordination to the indium center. These effects will be examined in future studies.

## Supplementary Information

Below is the link to the electronic supplementary material.

Supplementary Material 1

## Data Availability

Data are available on reasonable requests.
