# Supplementary material for: Determination of water in organic solvents by ligand-exchange reaction of Tris(2-methyl-8-quinolinolato)indium(III) complex
Source: Anal Sci. 2026 Apr 20;42(6):479–86. doi: 10.1007/s44211-026-00911-3 (PMC13201274; doi:10.1007/s44211-026-00911-3)
Supplement: Supplementary file 1 — Supplementary Material 1 [file 44211_2026_911_MOESM1_ESM.docx]

Supplementary Information

Note

**Determination of Water in Organic Solvents**

**by ligand exchange reaction of tris(2-methyl-8-quinolinolato)Indium(III) Complex**

Nanami Watanabe, Nobuo Uehara, and Arinori Inagawa*

*School of Engineering, Utsunomiya University, 7-1-2, Yoto, Utsunomiya, Tochigi, 321-8585, Japan*

Corresponding to ainagawa@a.utsunomiya-u.ac.jp (A. Inagawa)

Table S1. Water content in organic solvent determined by Karl-Fisher titration

| Ethanol | | | Ethyl Acetate | | | 1-octanol | | |
| --- | --- | --- | --- | --- | --- | --- | --- | --- |
| Sample No. | Concentration, %w/w | Concentration/mol L^-1^ | Sample No. | Concentration, %w/w | Concentration/mol L^-1^ | Sample No. | Concentration, %w/w | Concentration/mol L^-1^ |
| blank | 0.4863 | 0.2133 | blank | 0.201 | 0.1006 | blank | 0.3084 | 0.1426 |
| 1 | 6.6444 | 2.9137 | 1 | 0.6889 | 0.3449 | 1 | 0.9636 | 0.4456 |
| 2 | 12.0653 | 5.2908 | 2 | 1.0796 | 0.5405 | 2 | 1.5407 | 0.7124 |
| 3 | 16.9492 | 7.4325 | 3 | 1.5089 | 0.7555 | 3 | 2.0453 | 0.9457 |
| 4 | 21.1585 | 9.2783 | 4 | 1.86 | 0.9313 | 4 | 2.6663 | 1.2329 |


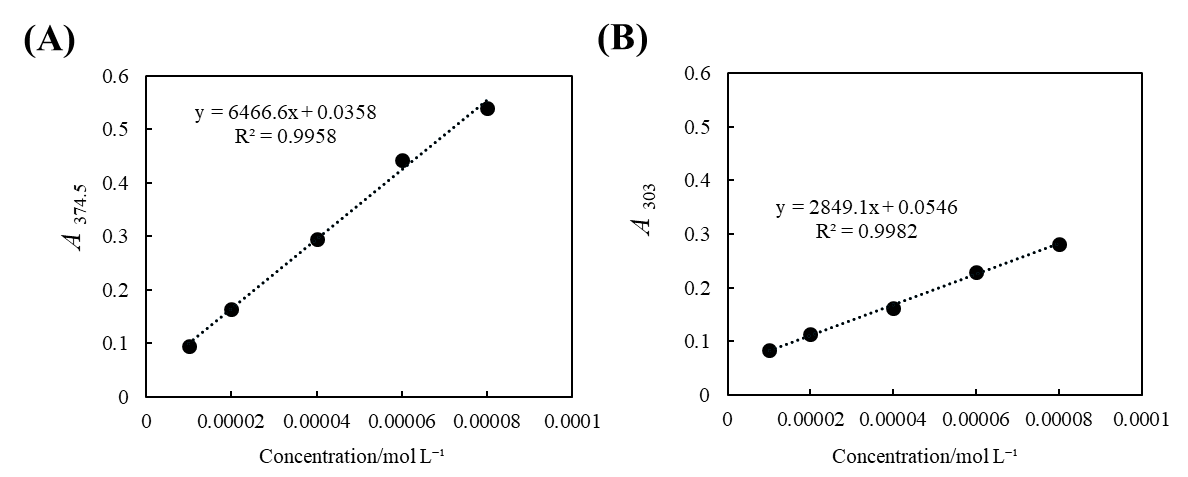


Figure S1. Determination curve of In(MQ)3 and HMQ in ethanol.


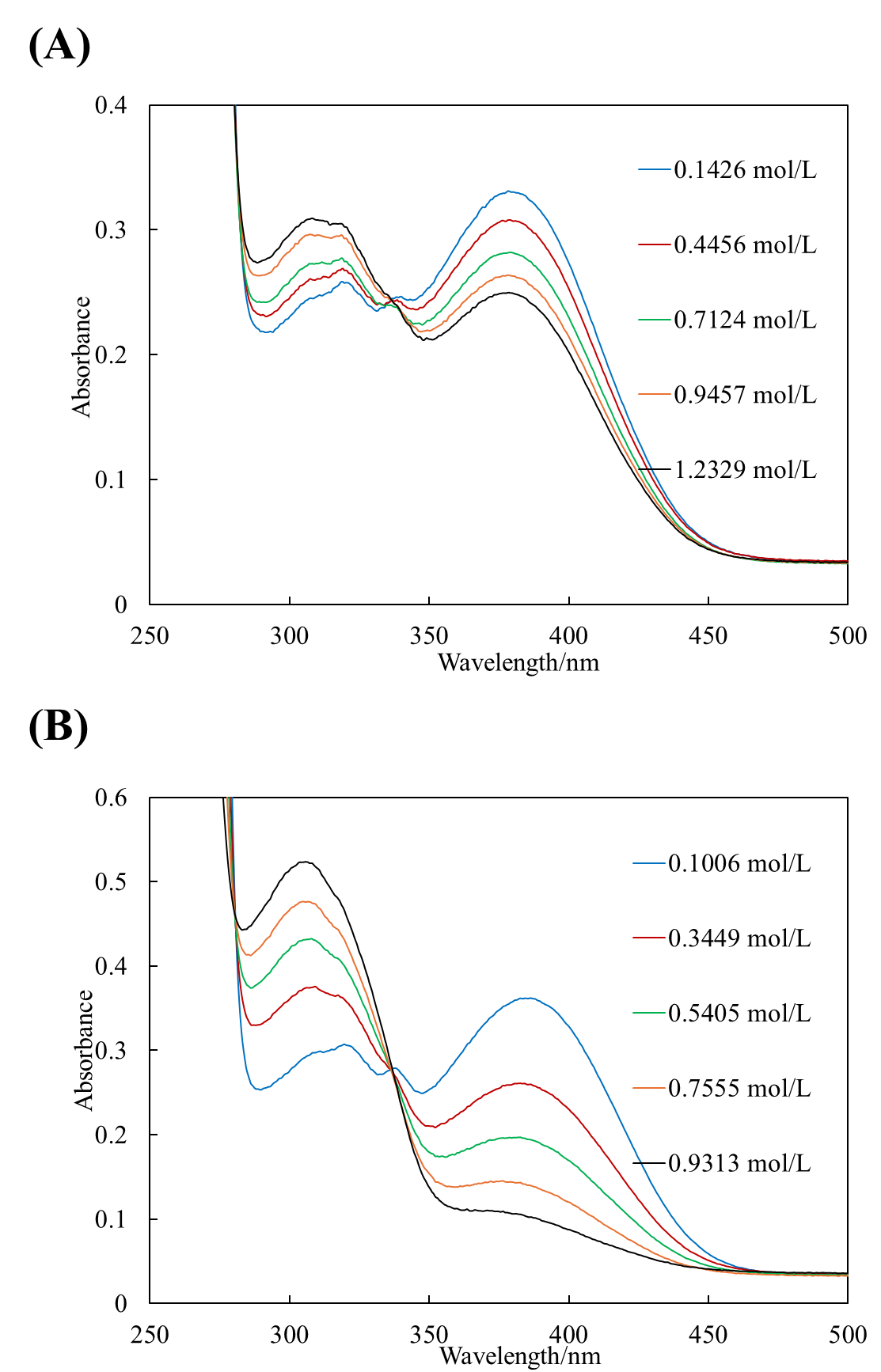


Figure S2. A series of absorption spectra of In(MQ)3 in (A)1-octanol and (B) mixture of ethyl acetate and dichloromethane containing a given concentration of water.


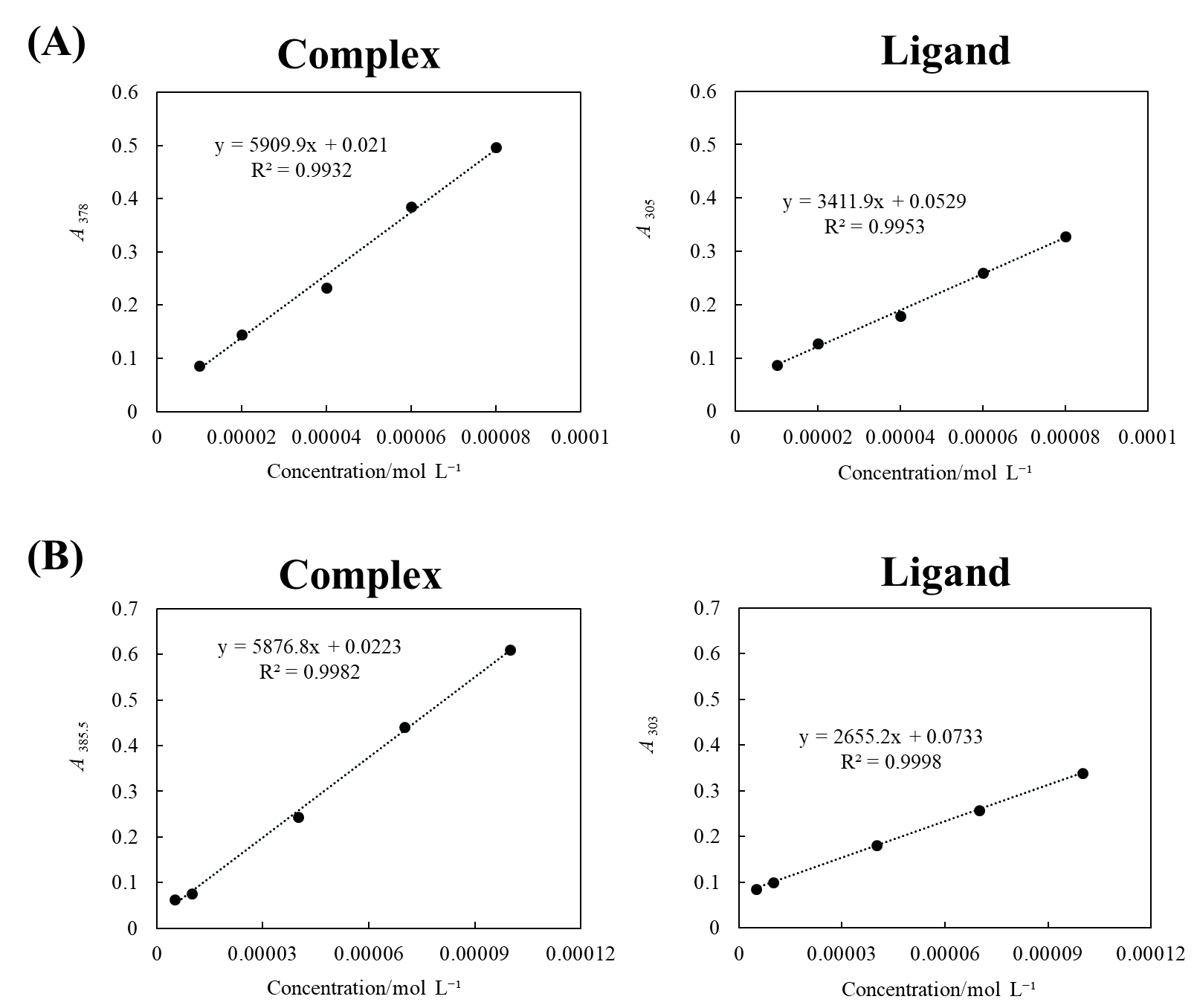


Figure S3. Determination curve of In(MQ)3 and HMQ in dried (A) mixture of 1-octanol and dichloromethane, and (B) mixture of ethyl acetate and dichloromethane.
